# PPIRank - an advanced method for ranking protein-protein interations in TAP/MS data

**DOI:** 10.1186/1477-5956-11-S1-S16

**Published:** 2013-11-07

**Authors:** Xiaoyun Sun, Pengyu Hong, Meghana Kulkarni, Young Kwon, Norbert Perrimon

**Affiliations:** 1 Department of Computer Science, Brandeis University, Waltham, MA USA; 2 Department of Genetics, Harvard Medical School, Boston, MA USA

**Keywords:** Protein-Protein Interaction, TAP/MS, Spectral Counts

## Abstract

**Background:**

Tandem affinity purification coupled with mass-spectrometry (TAP/MS) analysis is a popular method for the identification of novel endogenous protein-protein interactions (PPIs) in large-scale. Computational analysis of TAP/MS data is a critical step, particularly for high-throughput datasets, yet it remains challenging due to the noisy nature of TAP/MS data.

**Results:**

We investigated several major TAP/MS data analysis methods for identifying PPIs, and developed an advanced method, which incorporates an improved statistical method to filter out false positives from the negative controls. Our method is named PPIRank that stands for **PPI rank**ing in TAP/MS data. We compared PPIRank with several other existing methods in analyzing two pathway-specific TAP/MS PPI datasets from *Drosophila*.

**Conclusion:**

Experimental results show that PPIRank is more capable than other approaches in terms of identifying known interactions collected in the BioGRID PPI database. Specifically, PPIRank is able to capture more true interactions and simultaneously less false positives in both Insulin and Hippo pathways of *Drosophila Melanogaster*.

## Background

Protein-protein interactions (PPIs) are fundamentally characterized in many biological processes, such as gene expression, cell growth, proliferation and the regulatory complex formation. Information acquired from PPI data has two important features: definitive (i.e., direct interactions between proteins) and quantitative (i.e., the strength of PPI may vary). Hence identifying all functional PPIs is important not only for the understanding of the structure and function of biological systems, but also for the construction of reliable networks [1,2].

There are three widely-used biochemical technologies for identifying PPIs: the classic Co-ImmunoPrecipitation (Co-IP) and yeast two-hybrid [3] assays, and the more recent tandem affinity purification (TAP) coupled with mass-spectrometry (MS) [4-6]. Co-IP is usually used to identify interactions between specific proteins. It is carried out by immunoprecipitation with an antibody against a specific protein followed by checking the presence of other proteins in the immune complex. Although Co-IP is simple and straightforward, it only deals with limited number of proteins, thus lacks proteome capacity. On the other hand, yeast two-hybrid and TAP/MS assays are high-throughput, they can be used to explore genome-wide interaction partners. Yeast two-hybrid screen involves transfection of mutant yeast strains with separate bait and prey plasmids, where the bait is the protein of interest, and preys are proteins interact with the bait protein, and are typically screened from a library of genes. The interaction between bait and prey is identified by the growth of the yeast strain under selective conditions. While yeast two-hybrid is quite powerful, it is known to be false-positive prone. In addition, the interactions detected from a heterogeneous context (for species other than yeast) do not always occur endogenously. During recent years, TAP/MS has evolved to be a prevail technique to study endogenous PPIs. It has been frequently used in large-scale to identify novel PPIs under physiologically relevant conditions in a variety of cells or multi-cellular organisms [7-11]. Another advantage of TAP/MS technique is that it can be combined with other quantitative proteomics approaches to characterize the dynamics of protein-complex assembly [12,13].

**Figure 1:**
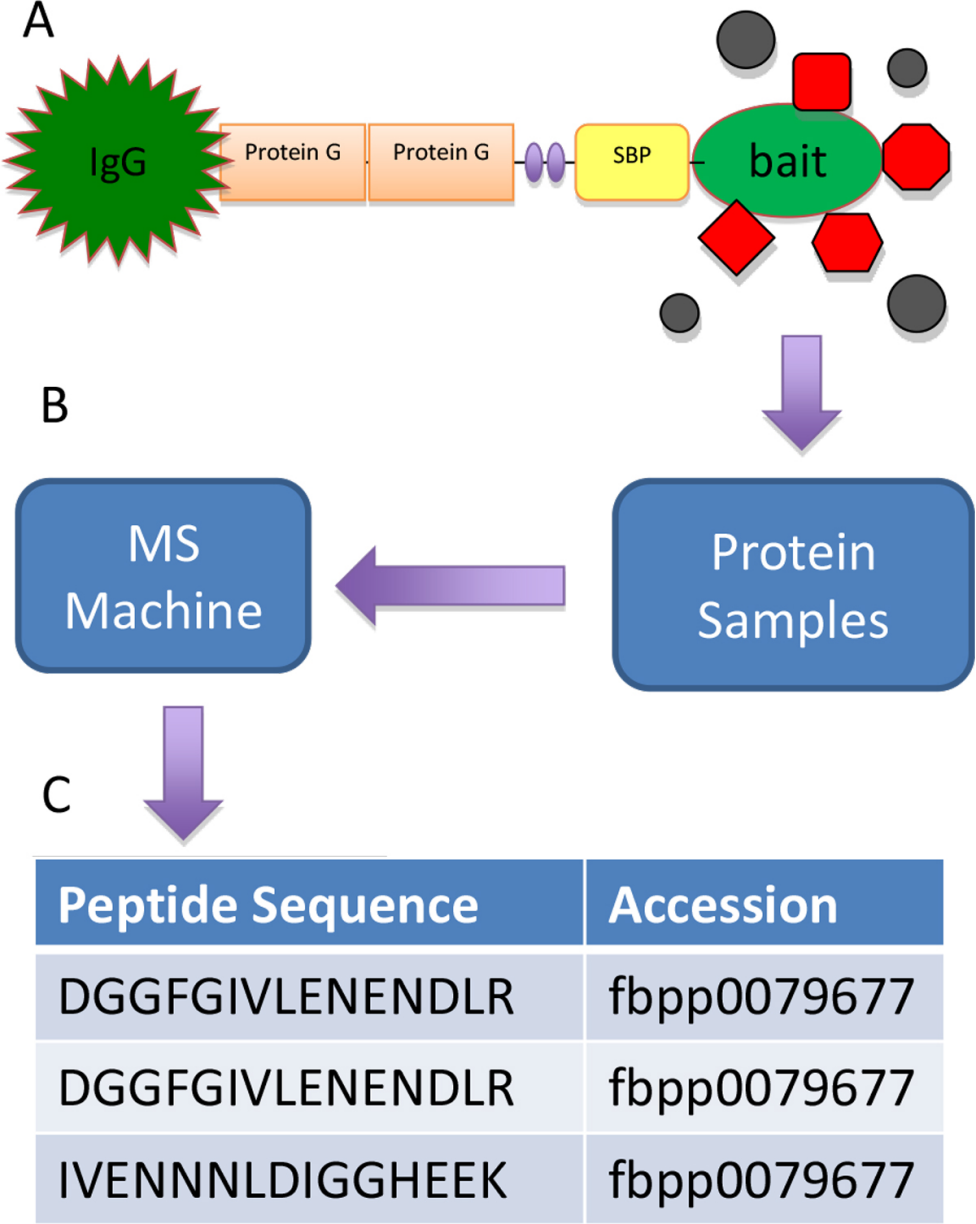
**Overview of the tandem affinity purification coupled with mass-spectrometry (TAP/MS) technology**. (A) A tagged protein is pulled down via its tag, together with associated proteins (red) and other non-specific interacting proteins (black). (B) The protein samples collected are then broken down into peptides by proteases and analyzed by mass-spectrometry. (C) A list of peptide sequences and corresponding proteins from each sample are reported as the results.

TAP/MS technique is composed of two essential components: TAP and MS (Figure 1). TAP efficiently isolates native protein complexes from cells, which are then digested by proteases into peptides. The peptides are identified by MS. To study PPIs specific to a particular signaling pathway, a set of cell lines need to be generated, with each of them stably expresses a TAP-tagged version of one of the core proteins in the signaling pathway. Similar to yeast two-hybrid screen, the tagged proteins are called baits, and the proteins interacting with the bait proteins are called preys. Tagging of the baits rarely affect their function, but greatly facilitates the isolation of bait-prey complexes for the follow-up MS analysis. The tagged cells can be treated with the desired stimuli followed by protein extraction to produce cell lysates. The lysates are then incubated with affinity purification beads, where the TAP-tagged protein is pulled down via its tag, together with its true interactors and other proteins retained through non-specific binding (these proteins contribute to most of the false positives). The collected protein samples are then broken down into peptides by proteases and subsequently analyzed by MS to reveal the identity and abundance of these peptides. The TAP/MS data derived from the tagged cell lines are called the bait purifications. In addition to these special cell lines, a negative control cell line, which either not expressing any TAP-tagged protein or expressing an un-related protein, for example, green fluorescent protein (GFP), is often included in the same study. The control cells should be processed the same way as specific cell lines, it is important to use these control samples to filter out non-specific interactors.

MS is a powerful approach to determine protein/peptide identity based on the mass-to-charge ratios. It has been widely used to determine the composition of protein complexes [4-6][12,13]. An important step in processing MS data is to identify the source proteins giving rise to the small peptides identified by MS. Only peptides with unique mapping to specific proteins are retained, peptides mapped to multiple proteins are eliminated in this step to reduce ambiguities. We can roughly quantify the abundance of a protein by calculating the number of unique peptides mapped to it. Numerous studies have shown that MS quantification is quite reliable. At the same time, MS data is also noisy, partially due to the high sensitivity of this technology. To improve the reliability in PPI identification by MS, multiple replicates are recommended for each cell line per experimental condition, so that reproducible true-interactors can be identified.

TAP/MS technology has been applied to study many distinct biological problems. A number of large-scale pathway specific TAP/MS PPI datasets have been generated, which are highly valuable for the configuration of the signaling networks. Computational analysis of these datasets is essential for this purpose. However, currently available analysis tools are not sophisticated enough, as high false-positive and false negative rates are commonly associated with TAP/MS data analysis. Therefore, better algorithms are needed to improve TAP/MS data analysis, so that lower false-positive rate and increased sensitivity to capture true interactions can be achieved.

Initial methods for TAP/MS PPI data analysis only score the presence or absence of proteins (thus the binary information) [14]. Recent methods implement quantitative features such as the number of peptides of a protein detected in MS, also named as spectral counts (SCs). The abundance of a protein detected in the MS is reflected by its SC. Currently, there are three major methods for the analysis of TAP/MS PPI data: Normalized Spectral Abundance Factor (NSAF) method [15], Comparative Proteomic Analysis Software Suite (CompPASS) method [16], and Significance Analysis of Interactome (SAINT) [17] method. They all utilize the quantitative feature of MS, but take to different level of consideration of negative control, frequency of detection and experimental reproducibility.

NSAF calculates the abundance of the prey in the purification by normalizing the number of its peptides to the length of the prey and the total number of the peptides. CompPASS calculates *Z *score (*Z*_*SC*) and *D *score (*D*_*SC*) for each bait-prey pair in each purification. Z_SC is calculated using the mean and standard deviation of the spectral counts of the prey across all purifications. *D*_*SC *takes into account both the reproducibility and the frequency of each observed prey with different baits. SAINT uses a Bayesian approach to estimate the probability of true interaction between prey and bait. For all these methods, the average score is assigned to each prey-bait pair if multiple replicates are available.

NSAF, CompPASS and SAINT can effectively analyze many datasets [15]. However, there is still room for improvements. For example, although *NSAF *is simple to compute, it does not utilize negative controls, which are valuable to reduce false positives. CompPASS performs well for datasets having large number of unrelated baits. But if all baits belong to the same pathway, some true interactors with higher detection frequency will be filtered out as sticky proteins. In addition, CompPASS is designed to utilize duplicates at maximum, which prevents it from sufficiently taking advantages of more replicates to better identify PPIs. It is shown that duplicates are far from enough to achieve saturated sampling of complex protein mixtures [18]. On the other hand, SAINT over-penalizes true but "appear" to be not reproducible interactions (e.g. interactions have high average SC, however, are not captured in all replicates). SAINT averages the posterior probability of interactions in all replicates; no detection of a PPI in one replicate will significantly reduce the average score. Therefore, SAINT can miss some statistically-significant PPIs that are not necessarily 100% reproducible. Recently we reported our effort of developing a new algorithm for TAP/MS PPI data analysis [19]. In this paper we conducted more detailed analysis of two *Drosophila *pathway-specific TAP/MS datasets with this algorithm (renamed PPIRank), and also performed parallel comparisons with other methods. PPIRank consistently scores PPIs from TAP/MS experiments with high accuracy and reduced false positives.

## Results

As reported before [19], we developed a new protein-protein interactions ranking (PPIRank) method for TAP/MS PPI data analysis. PPIRank quantifies PPI from spectral counts, taking into account of negative control, experimental reproducibility and variation with improved statistical analysis. PPIRank outperformed existing methods in capturing true interactions with higher specificity [19]. To characterize this method in more detail, we performed a comprehensive analysis of two pathway-specific TAP/MS PPI datasets from *Drosophila Melanogaster*: the Insulin and the Hippo pathway.

**Figure 2:**
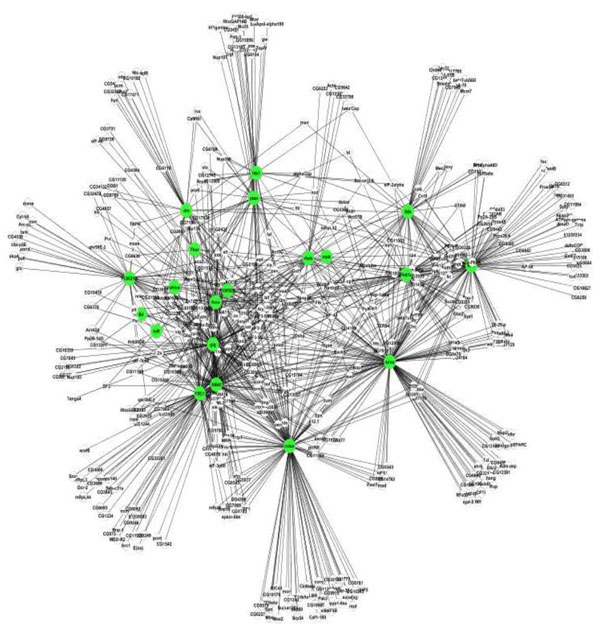
**A map of predicted protein-protein interactions (PPIs) in the Insulin pathway computed by PPIRank**. Bait proteins are highlighted in green.

**Figure 3:**
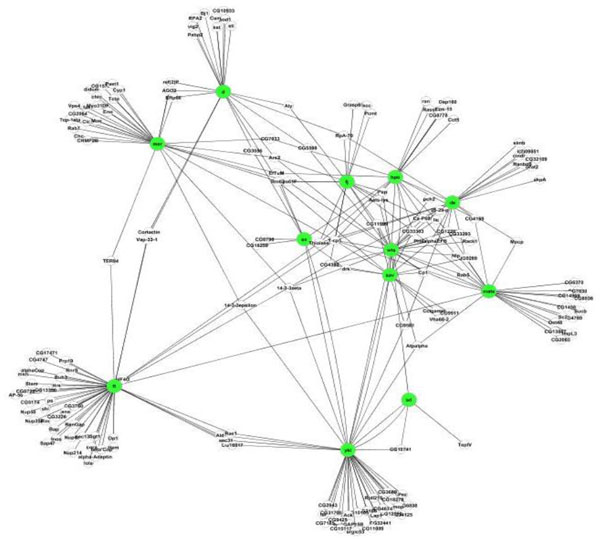
**A map of predicted PPIs in the Hippo pathway computed by PPIRank**. Bait proteins are highlighted in green.

PPIRank identified a total of 1419 interactions between 509 proteins in the Insulin dataset and 286 interactions between 191 proteins in the Hippo dataset, respectively. These interactions significantly expanded the components in both pathways. After filtering out heat shock proteins and ribosomal proteins, we obtained 1080 interactions in the Insulin pathway (Figure 2) and 255 interactions in Hippo pathway (Figure 3).

**Figure 4:**
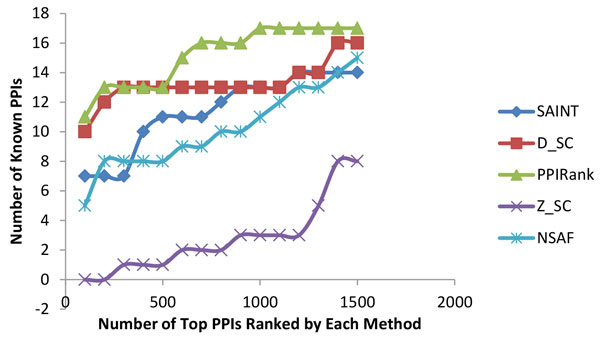
**Compare the performances of five algorithms on the Insulin dataset**. PPIRank (in green) was able to consistently report more known interactions than other approaches.

**Figure 5:**
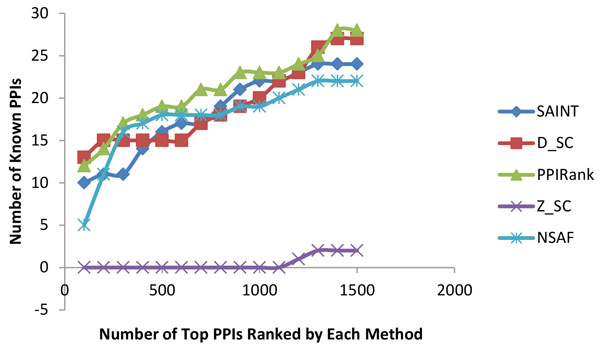
**Compare the performances of five algorithms on the Hippo dataset**. PPIRank (in green) was able to consistently report more known interactions than other approaches.

We performed a number of tests to evaluate PPIRank results: first, we examined whether PPIRank can identify more known interactions than other approaches. 27156 known interactions in *Drosophila *were collected from BioGRID PPI database (http://www.thebiogrid.org/). Effort was made to choose only physical interactions but not genetic interactions, as indirect genetic interactions can give rise to false positives. The overlaps between the top-scored interactions by each algorithm and the known interactions were counted and shown in Figure 4 and Figure 5. Interactions identified by PPIRank consistently showed the highest overlap with known interactions, demonstrating that PPIRank is more comprehensive compared to other methods.

Second, we performed a side-by-side comparison of data handling between PPIRank and SAINT, as it was developed relatively recent [17]. Table 1 lists the canonical interactions in the Insulin pathway detected at different time points by PPIRank and/or SAINT. Equivalent thresholds were used in PPIRank and SAINT to score significant interactions. PPIRank, but not SAINT, is able to capture the interactions *rictor - Sin1 *(30 min), *TSC - gig *(0 and 30 min), and *InR - chico *(0 min). These examples demonstrate that PPIRank has a reasonable balance between scoring and penalizing, while the stringent penalizing non-reproducibility in purifications and sporadic appearances in controls in SAINT leads to the failure of detection of these interactions.

**Table 1:** Canonical interactions in Insulin pathway scored by PPIRank and SAINT.

bait	prey	PPIRank	SAINT	Time	SCs
*chico *	*14-3-3epsilon *	Y	Y	0	25, 21, 21

*chico *	*14-3-3epsilon *	Y	Y	10	53, 50, 50
*chico *	*14-3-3zeta *	Y	Y	0	8, 13, 15,

*chico *	*14-3-3zeta *	Y	Y	10	34, 20, 32

*dm *	*Max *	N	N	0	0, 0, 1

*gig *	*TSC1 *	Y	Y	0	5, 8, 3

*gig *	*TSC1 *	Y	Y	10	3, 4, 5

*gig *	*TSC1 *	Y	N	30	8, 3, 0

*TSC1 *	*gig *	Y	N	0	4, 0, 4

*TSC1 *	*gig *	Y	Y	10	7, 5, 4

*TSC1 *	*gig *	Y	N	30	12, 12, 0

*InR *	*chico *	Y	N	0	3, 21, 0

*InR *	*chico *	Y	Y	10	19, 17, 16, 7

*InR *	*chico *	Y	Y	30	12, 6

*chico *	*InR *	Y	Y	0	17, 21, 21

*chico *	*InR *	Y	Y	10	8, 8, 9

*lkb1 *	*Mo25 *	Y	Y	0	16, 3, 1

*lkb1 *	*Mo25 *	N	N	10	0, 1, 3

*Pi3K21B *	*chico *	Y	Y	0	11, 8, 3

*Pi3K21B *	*chico *	Y	Y	10	18, 25, 18

*Pi3K21B *	*chico *	Y	Y	30	25, 31, 3

*chico *	*Pi3K21B *	Y	Y	10	2, 3, 9

*Pi3K92E *	*Pi3K21B *	Y	Y	0	41, 13, 33

*Pi3K92E *	*Pi3K21B *	Y	Y	10	38, 32, 31

*Pi3K92E *	*Pi3K21B *	Y	Y	30	52, 2, 5

*Pi3K21B *	*Pi3K92E *	Y	Y	0	34, 39, 2

*Pi3K21B *	*Pi3K92E *	Y	Y	10	56, 57, 52

*Pi3K21B *	*Pi3K92E *	Y	Y	30	51, 58, 69

*rictor *	*Sin1 *	Y	N	30	0, 3, 3

*S6kII *	*RpS6 *	Y	Y	0	4, 6, 7

*S6kII *	*RpS6 *	Y	Y	10	8, 5, 6

*S6kII *	*RpS6 *	Y	Y	30	8, 8, 11

*Thor *	*eIF-4E *	Y	Y	0	60, 64, 40

*Thor *	*eIF-4E *	Y	Y	10	39, 52, 45

*Thor *	*eIF-4E *	Y	Y	30	60, 34, 46

Equivalent threshold used to select same number of significant interactions in each method. Three time points are shown here (0, 10, and 30 minutes). Y = significant interactions, N = non-significant interactions.

**Figure 6:**
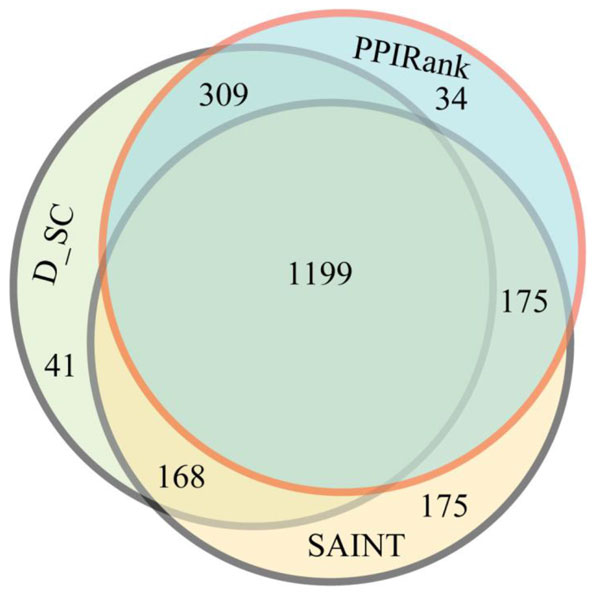
**Distribution of significant interactions in the insulin pathway scored by PPIRank, SAINT and CompPASS D_SC**. 1199 out of 1717 interactions are identified by all the three approaches. Each approach can score overlapping but distinct list of interactions.

**Figure 7:**
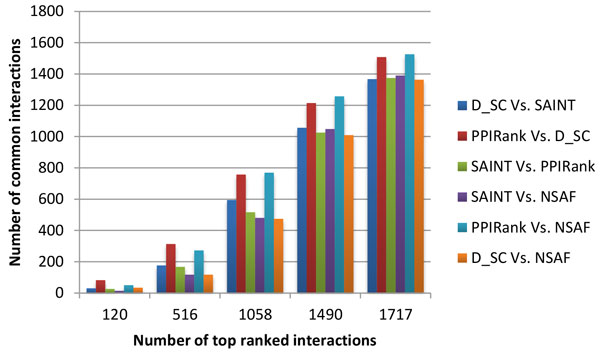
**Pairwise analysis profile of the Insulin pathway by different approaches**. TAP/MS data from the Insulin pathway study were analyzed by 4 different methods. Increasing number of the top ranked interactions were selected, and common interactions were compared pairwisely.

**Figure 8:**
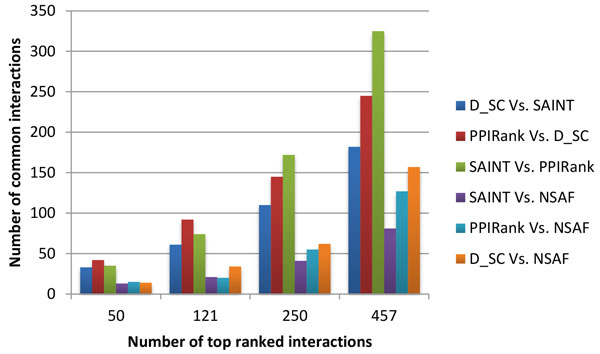
**Pairwise analysis profile of the Hippo pathway by different approaches**. The analysis is performed the same as described in Fig. 7.

Finally, we performed an extensive comparison of commonly identified interactions by PPIRank and other approaches. The distribution of the top 1717 interactions in the Insulin pathway analyzed by PPIRank, SAINT and *D_SC *approaches is shown in Figure 6. Different approaches yield overlap but distinct results, with 1199 out of 1717 (70%) interactions are common, and others are either partially or not shared, which largely determine the quality of different methods. We noted that 98% of PPIs identified by PPIRank or D_SC are also scored by at least one other method, while the number is 90% for SAINT. We further examined the overlapping interactions identified by different approaches in detail by comparing the results of both the Insulin and Hippo pathways by PPIRank, SAINT, *D_SC *and also NSAF (*Z_SC *is not considered because of its poor performance as shown in Figures 4 and Figure 5). Increasing number of top ranked interactions by each method was selected and compared. The number of the common interactions identified by any two methods was calculated and plotted in Figure 7 and Figure 8. PPIRank consistently has the highest number of common interactions with other approaches (Figure 7 and 8).

## Conclusions and discussions

With two independent datasets, we have shown that PPIRank outperformed other methods with improved accuracy. Significantly, PPIRank is able to detect real and important interactions with relatively fewer false-positives. There are a number of improvements in PPIRank compared to other methods. For example, PPIRank will filter out interactions with only one SC in each of the three replicates, as single SC is prone to experiment error or noise. SAINT, on the other hand, will consider such an interaction significant because it appears in all replicates. PPIRank differs with SAINT in dealing with PPIs with significantly differential SCs between bait purifications and negative controls: PPIRank assigns a high score to a PPI that has high SCs in all replicates while has much lower SCs in negative controls.

PPIRank can also "rescue" some interactions (false negative) that seem to be non-reproducible (mainly due to experimental noise), but are statistically significant. For instance, a known interaction *InR - chico *has SC = 3/21/0 in three replicates (TABLE I). It was not scored by SAINT due to the lack of reproducibility. PPIRank considered this interaction as significant since it was not detected in 6 control replicates and had relatively high SCs in two replicates, thus appeared to be statistically significant.

PPIRank also identified a number of the Brahma complex proteins interact with components of the Insulin pathway. Brahma protein complex is also called SWI/SNF chromatin-remodeling complex and is associated with transcriptional regulator for the remodeling of chromatin structure during transcription [20]. A number of Brahma complex proteins (MBD-R2, dalao, brm, mor and Bap55) were identified by PPIRank to interact with major components in the Insulin pathway (rictor, TSC1, sima, foxo, S6KII and gig). Interestingly, these interactions are enhanced by insulin treatment. It is significant that a recent report confirmed our finding, and the interaction between S6KII and dalao was verified by Co-IP experiments [21].

In summary, we developed and vigorously tested PPIRank, an advanced statistical method for TAP/MS data analysis. PPIRank is based on label-free quantification by spectral counts and incorporates statistical analysis of frequency and reproducibility of observed interactors across all biological replicates. One major advance of PPIRank is the inclusion of the false positive rate of interactors interpreted from statistical *p*-values to filter out non-specific interactors. PPIRank has been successfully applied to the analysis of TAP/MS datasets of *Drosophila *Insulin and Hippo pathways. The result shows that PPIRank is capable of identifying more known interactions than other existing approaches and produces high quality results. The statistical analysis of PPIRank requires negative controls and at least three biological replicates. While these demands lead to higher experimental cost, we argue that they are necessary for generating high-confidence results.

## Methods

The scoring function of PPIRank is based on NSAF and CompPASS *D*_*SC*. Here, NSAF estimates the relative abundance of the *j*-th prey in a purification of the *i*-th bait as in equation (1):

(1) $$NSAF_{i,j}=\frac{\frac{SC_{i,j}}{L_{j}}}{\sum_{k=1}^{N}\frac{SC_{j,k}}{L_{k}}}$$

Where *SC_i,j _*and *L,_j _*are the spectral count and the length of the *j*-th prey, respectively, and *N *is the number of preys in purification of the *i*-th bait. The *NSAF *score is calculated for each prey-bait pair in a purification.

CompPASS computes a weighted *D*_*SC *of the *j*-th prey of the *i*-th bait as:

(2) $$WD\text{\_}SC_{i,j}=\sqrt{SC_{i,j}{\left(\frac{K}{\sum_{i-1}^{K}f_{i,j}}W_{j}\right)}^{N}}$$

where *f*_*i,j *_= 1 if the *j*-th prey is identified in the purification of the *i*-th bait, otherwise *f*_*i,j *_= 0; *N *is the number of replicates in which the interaction is detected; and *K *is the total number of purifications. The summation of *f*_*i,j *_indicates the total number of occurrences of an interaction in all purifications. The ratio between *K *and the summation of *f*_*i,j *_is the frequency of the *j*-th prey being observed across all purifications. *W*_*j *_= *std*_*j*_/*mean*_*j *_if *std*_*j*_/*mean*_*j *_> 0, otherwise *W*_*j *_= 1. CompPASS believes that *std*_*j *_is more likely to be higher than *mean*_*j *_if the *j*-th prey is a true interactor of the *i*-th bait. The weight factor *W*_*j *_is a multiplicative factor designed to award preys that have large variance of SCs and been frequently detected in all purifications.

PPIRank was described in detail in another study [19], it is an improvement over those of NSAF and CompPASS. First, a new defined reproducible term *R *was introduced. The difference between the reproducibility term *N *used in CompPASS (see equations 2) and PPIRank is that we calculate *R *as *N *divided by the total number of biological replicates *T*. Our definition of the *R *term is relative to the total number of replicates, and is therefore a more appropriate indicator for experimental reproducibility. Taking consideration of the reproducibility factor, we apply *R *to the power of the weighted frequency term:

(3) $${\left(W_{j}\times \frac{K}{\sum_{i=1}^{K}f_{i,j}}\right)}^{R}$$

Second, PPIRank utilizes negative controls in a more meaningful way. Negative controls are great resource for filtering non-specifically PPISs in the TAP/MS samples. SAINT uses a complicated method to estimate the spectral count distribution for false interactions from negative controls. Here, we compute a false discovery rate (FDR) for each observed prey using negative controls, which can not only be used to help filter out the majority of false positives, but also rescue some interactors that are abundant in bait purifications while having very low SCs in negative controls (most likely noise in negative controls). Using the method proposed in [22], we can compute the FDR of a prey *j *as:

(4) $$FDR_{i,j}=\frac{1}{1+\frac{1}{e\times p_{i,j}\times \text{log}\left(p_{i,j}\right)}}$$

where *e *is the base of the natural logarithm, and *p_i,j _*is the *p*-value generated by applying the rank-sum test to compare two populations: the SCs of *j*-th prey in the purifications of *i*-th bait *vs *the SCs of *j*-th prey in the negative controls. Finally, we have the following function to score any given bait-prey pair (*i, j*) given its MS data:

(5) $$PPIRank_{i,j}=NSAF_{i,j}\times {\left(1-FDR_{i,j}\right)}^{T}\times {\left(\frac{W_{j}\times K}{\overset{i=K}{\underset{i=1}{\sum }}f_{i,j}}\right)}^{R}$$

where *NSAF_i,j _*is the averaged NSAF score if there are multiple replicates available.

The PPIRank score of a bait-prey pair depends on the total number of TAP/MS samples, the number of replicates, and the SC of the prey in each individual replicate. We can rank the bait-prey pairs in a dataset by their PPIRank scores. The higher the score, the more significant the interaction is. We recommend users to set appropriate PPIRank score cutoffs through the reference of known interactions.

## Competing interests

The authors declare that they have no competing interests.

## Authors' contributions

Xiaoyun Sun and Pengyu Hong designed the algorithm and wrote the manuscript. Xiaoyun implemented and tested the algorithm. Meghana Kulkarni, Young Kwon and Norbert Perrimon contributed the data used by this work and participated into the discussions. All authours reviewed and approved the manuscript.

## Declarations

The publication costs for this article were funded by the corresponding author.

This article has been published as part of *Proteome Science *Volume 11 Supplement 1, 2013: Selected articles from the IEEE International Conference on Bioinformatics and Biomedicine 2012: Proteome Science. The full contents of the supplement are available online at http://www.proteomesci.com/supplements/11/S1.
